# Directed midbrain and spinal cord neurogenesis from pluripotent stem cells to model development and disease in a dish

**DOI:** 10.3389/fnins.2014.00109

**Published:** 2014-05-20

**Authors:** Ilary Allodi, Eva Hedlund

**Affiliations:** Department of Neuroscience, Karolinska InstitutetStockholm, Sweden

**Keywords:** stem cells, *in vitro* neuronal networks, dopamine neuron, motor neuron, Parkinson disease, amyotrophic lateral sclerosis

## Abstract

Induction of specific neuronal fates is restricted in time and space in the developing CNS through integration of extrinsic morphogen signals and intrinsic determinants. Morphogens impose regional characteristics on neural progenitors and establish distinct progenitor domains. Such domains are defined by unique expression patterns of fate determining transcription factors. These processes of neuronal fate specification can be recapitulated *in vitro* using pluripotent stem cells. In this review, we focus on the generation of dopamine neurons and motor neurons, which are induced at ventral positions of the neural tube through Sonic hedgehog (Shh) signaling, and defined at anteroposterior positions by fibroblast growth factor (Fgf) 8, Wnt1, and retinoic acid (RA). *In vitro* utilization of these morphogenic signals typically results in the generation of multiple neuronal cell types, which are defined at the intersection of these signals. If the purpose of *in vitro* neurogenesis is to generate one cell type only, further lineage restriction can be accomplished by forced expression of specific transcription factors in a permissive environment. Alternatively, cell-sorting strategies allow for selection of neuronal progenitors or mature neurons. However, modeling development, disease and prospective therapies in a dish could benefit from structured heterogeneity, where desired neurons are appropriately synaptically connected and thus better reflect the three-dimensional structure of that region. By modulating the extrinsic environment to direct sequential generation of neural progenitors within a domain, followed by self-organization and synaptic establishment, a reductionist model of that brain region could be created. Here we review recent advances in neuronal fate induction *in vitro*, with a focus on the interplay between cell intrinsic and extrinsic factors, and discuss the implications for studying development and disease in a dish.

## Introduction

Induction of specific neuronal fates is restricted temporally and spatially in the developing central nervous system (CNS) through the coordinated integration of extrinsic morphogen signals and intrinsic determinants. The morphogens impose regional characteristics on neural progenitors at early developmental stages in a concentration-dependent fashion and establish distinct progenitor domains. Such domains are defined by unique expression patterns of transcription factors, which determine neuronal fate. These processes of neuronal fate specification can be recapitulated *in vitro* using pluripotent stem cells derived from embryonic stem cells (ESCs) or induced pluripotents stem cells (iPSCs).

## Dopamine and oculomotor neuron development

**Figure 1:**
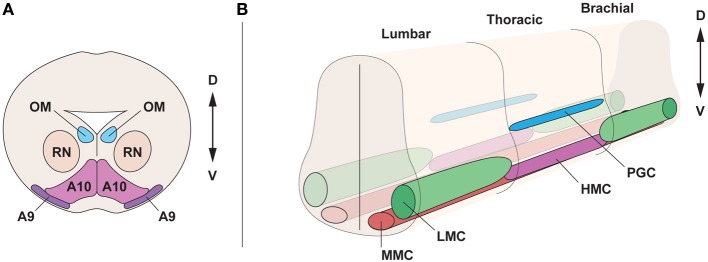
**Topographical organization of the midbrain and spinal cord in the embryo. (A)** Cross section of the midbrain, at E15.5, displaying oculomotor motor neurons (OM), innervating the ocular muscles, neurons of the red nucleus (RN) and dopamine neurons, subdivided into substantia nigra (A9) and ventral tegmental area (A10). **(B)** Motor neuron division in motor columns along the spinal cord at E13.5. Lateral motor column (LMC) motor neurons, innervating limb muscles, are present in brachial and lumbar segments. The preganglionic column (PGC), containing visceral motor neurons and the hypaxial motor column (HMC), innervating the abdominal walls, are present at thoracic levels. Medial motor column (MMC) motor neurons, innervating proximal muscles, are present all along the spinal cord. D, dorsal; V, ventral.

The developing ventral mesencephalon contains dopamine neurons of the substantia nigra pars compacta (SNc, A9 dopamine neurons) and the ventral tegmental area (VTA, A10 dopamine neurons), which are distinct in morphology, expression of G-protein-gated inwardly rectifying K^+^ channel 2 (Girk2) (Karschin et al., 1996), calbindin (Yamada et al., 1990; Liang et al., 1996; Liu et al., 1999), and Raldh1 (McCaffery and Drager, 1994; Chung et al., 2005; Jacobs et al., 2007) (Figure 1A). Moreover, they differ in their axonal projections and vulnerability to degeneration in Parkinson disease (Yamada et al., 1990; Damier et al., 1999a). Parkinson disease is characterized by cellular loss of SNc dopamine neurons that project to the striatum. Dopamine neurons of the VTA also degenerate, but to a lesser extent (Damier et al., 1999b). The developing midbrain also contains oculomotor neurons, which are general somatic efferents, connected with extraocular muscles and coordinating eye movement (Figure 1A). Oculomotor neurons are molecularly distinct from other somatic motor neurons (Hedlund et al., 2010) and require the transcription factor Phox2a (Pattyn et al., 1997), while lacking the transcription factor Hb9. Furthermore, oculomotor neurons are resistant to degeneration in the lethal diseases amyotrophic lateral sclerosis (ALS) and spinal muscular atrophy (SMA) (Gizzi et al., 1992; Kubota et al., 2000; Nimchinsky et al., 2000), which are characterized by the loss of somatic motor neurons that innervate muscles in arms, legs, trunk, and face.

**Figure 2:**
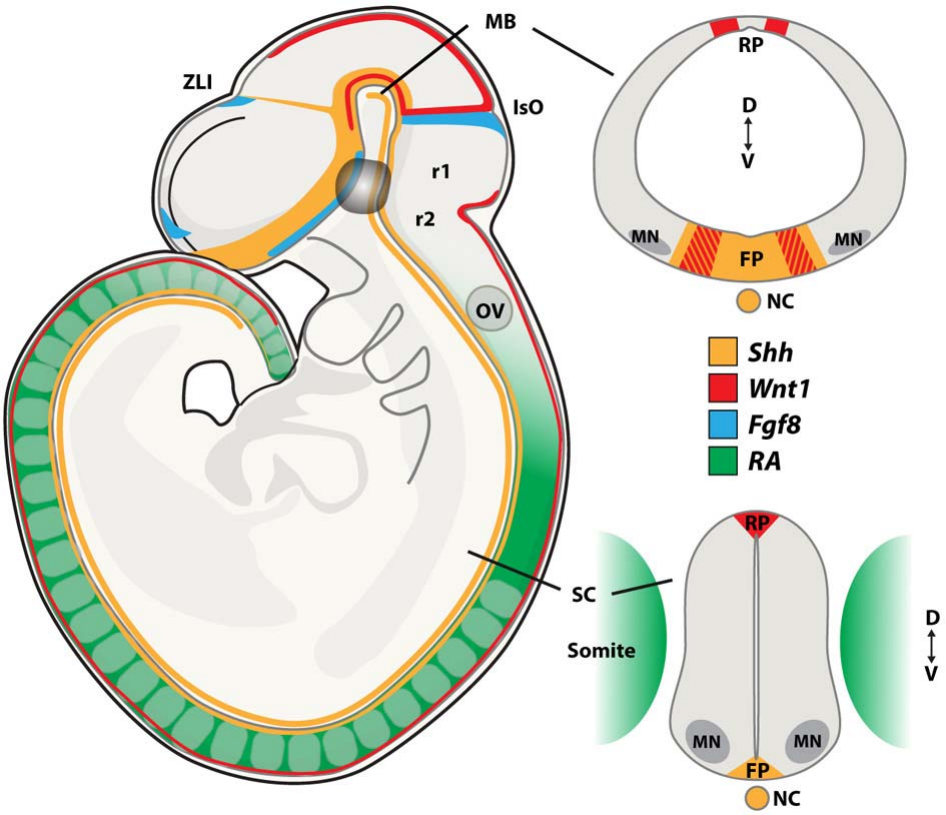
**Morphogen signaling during neural tube development**. During embryogenesis, neurons present in the midbrain (MB), including dopamine and oculomotor neurons are born at the intersection of the signaling molecules Shh, Wnt1, and Fgf8. Spinal motor neurons (MNs) are patterned by Retinoic Acid (RA) and Shh. Sagital and coronal views at the midbrain and spinal cord levels of the mouse embryo showing the expression patterns of these morphogens. FP, floor plate; IsO, isthmic organizer; MB, midbrain; NC, notochord; OV, otic vesicle; RP, roof plate; SC, spinal cord; ZLI, zona limitans intermedia; r1, rhombomer 1; r2, rhombomer 2; D, dorsal; V, ventral. Adapted from Aguila et al., 2012.

Dopamine neurons and oculomotor neurons are induced in the ventral neural tube of the midbrain through the actions of the morphogens Sonic hedgehog (Shh), fibroblast growth factor 8 (Fgf8), Wnt1 and retinoic acid (RA) (Figure 2). Midbrain dopamine neurons are generated from neuroepithelial cells of the floor plate that have a non-neurogenic character (Andersson et al., 2006; Ono et al., 2007). The floor plate is a specialized glial structure located in the most ventral midline of the neural tube from the midbrain to the tail region (Strahle et al., 2004). It controls neuronal subtype specification along the dorsoventral (D-V) axis through the secretion of Shh (Jessell, 2000). The capacity of the floor plate to give rise to neurons is restricted along the rostrocaudal axis of the brain, where floor plate cells located caudally to the midbrain do not normally give rise to neurons (Ono et al., 2007).

The isthmic organizer forms a boundary between the midbrain and hindbrain and controls patterning of the midbrain and the anterior hindbrain. It is essential for the specification and normal development of dopamine neurons and serotonin neurons (Brodski et al., 2003). Several signaling factors, including Shh, Fgf8, Fgf17, Fgf18, and Wnt1, are expressed by and around the isthmic organizer and are involved in this process (Figure 2). The combination of Shh and Fgf8 is necessary for the induction of dopamine neurons in the rostral forebrain and the lateral midbrain (Wang et al., 1995; Ye et al., 1998). However, Shh is no longer required after embryonic (E) day E10.5 in the mouse. At this developmental stage, Foxa2, a forkhead transcription factor, induced by Shh, is essential for the generation of midbrain dopamine neurons (Ferri et al., 2007). ChIP-seq analysis recently showed that Foxa2 positively regulated determinants of dopamine neurons, including the transcription factors Lmx1a, Lmx1b, Msx1, and Ferd31, while repressing ventrolateral genes in midbrain dopamine progenitors, including Helt, Tle4, Otx1, Sox1, and Tal2. Foxa2 also repressed the expression of Gli1, Gli2, and Gli3, which are the intracellular transducers of Shh signaling (Mavromatakis et al., 2011). Foxa2 has previously been shown to regulate the expression of the orphan transcription factor NR4A2 (Nurr1) (Ferri et al., 2007), which is required for the induction of a number of genes that confer a dopamine neuron transmitter phenotype, including TH, VMAT2, DAT, AADC, and c-ret (Wallen et al., 1999). In addition, Foxa2 appears to play a role in regulating axon trajectories around the midline through targets including Slit2 and 3 (Metzakopian et al., 2012). The transcription factors Lmx1a and Lmx1b are important for the specification of dopamine neurons (Smidt et al., 2000; Andersson et al., 2006). Importantly, dopamine progenitors in the developing midbrain can be subdivided into molecularly distinct medial and lateral domains and these show differential sensitivity to the loss of Lmx1a and Lmx1b. Here, Lmx1a converts non-neuronal floor plate cells into medial dopamine progenitors (Andersson et al., 2006; Ono et al., 2007). However, Lmx1a is not absolutely required for the generation of these neurons, since the deficiency in dopamine neurogenesis in the Lmx1a knockout mouse recovers over time (Deng et al., 2011). Lmx1b is required for generating lateral dopamine progenitors, that in fact do not appear to originate from the floor plate, and which are selectively ablated in Lmx1b mutants (Deng et al., 2011). Oculomotor neurons are generated immediately lateral to the dopamine progenitor domain from cells that express Lmx1b, Phox2a, Sim1, and Nkx6.1. The sequential generation of oculomotor neurons and red nucleus neurons seems controlled by Lmx1b, which is more broadly expressed than Lmx1a in the early developing midbrain, through the activation of Phox2a (Deng et al., 2011). Phox2a, in turn, is required and sufficient to promote oculomotor neuron fate (Pattyn et al., 1997; Hasan et al., 2010) and to suppress the generation of red nucleus neurons at early developmental stages. Sim1, which is co-expressed with Phox2a in this progenitor pool, likely contributes to the specification of red nucleus neurons, but its activity appears suppressed by Phox2a (Deng et al., 2011). Phox2b is expressed in oculomotor neurons and ectopic expression of Phox2b is sufficient to induce Phox2a^+^ cranial motor neurons in the spinal cord (Pattyn et al., 1997; Dubreuil et al., 2000). Furthermore, the migration of newly born oculomotor neurons appears regulated by the Shh-inducible homedomain trancription factor Nkx6.1 (Prakash et al., 2009).

During early development, starting at E9, Fgf8 is expressed by the isthmic organizer (Heikinheimo et al., 1994) and can mimic the isthmic activity (Crossley et al., 1996; Lee et al., 1997). Fgf8 appears to maintain normal development of the midbrain and hindbrain by regulating transcription factors such as engrailed-1 (En1), En2, and Pax5 (Liu et al., 1999). Furthermore, Fgf8 appears to regulate anteroposterior patterning of dopamine neurons in a cell-autonomous manner and is neccessary for maturation of dopamine neurons (Lahti et al., 2012). Fgf8 also directs the rostral growth of axons from midbrain dopamine neurons by inducing the repulsion factor semaphorin 3F (Yamauchi et al., 2009). The migration and process orientation of VTA and SNc dopamine neurons can also be regulated through the interaction of CXCR4 in Nurr1^+^ dopamine progenitors and neurons and Cxcl12 in meningeal cells surrounding the ventral midbrain (Yang et al., 2013).

Wnt1 expression precedes Fgf8, starting at E8.0 and is required for early midbrain development. During early somite stages, Wnt1 is broadly expressed in the presumptive mesencephalon, but following neural tube closure, the expression gradually becomes refined to a narrow band of cells located immediately rostral to the isthmus and the dorsal midline of the CNS (Parr et al., 1993) (Figure 2). While Wnt1 does not have isthmic-like activity, it is essential, and deletion results in loss of midbrain and cerebellar structures by E10 and a reduction in the number of midbrain dopamine neurons (McMahon and Bradley, 1990; Thomas and Capecchi, 1990; McMahon et al., 1992; Panhuysen et al., 2004). Wnt1 appears necessary for the development of midbrain dopamine neurons since Fgf8 and Shh fail to induce expression of tyrosine hydroxylase (TH) (the rate limiting enzyme in dopamine synthesis) and the homedomain transcription factor Pitx3 (which is normally selectively expressed in midbrain dopamine neurons) in the Wnt1 knockout mouse (Prakash et al., 2006). Wnt1 and Wnt5 double knockout mice showed a greater loss of dopamine progenitors and neurons than single mutants, indicating that Wnt1 and Wnt5a cooperate to promote midbrain dopamine neurogenesis (Andersson et al., 2013). Ectopic expression of Wnt1 in the posterior hindbrain can induce midbrain dopamine neurons through the activation of the transcription factor Otx2 and the repression of Gbx2 and Nkx2.2 and induction of dopamine markers (Prakash et al., 2006). If ectopic Wnt signaling is combined with restored Lmx1b levels, midbrain dopamine neurons can be generated at even more posterior levels of the hindbrain, but not in the spinal cord (Joksimovic et al., 2012). Interestingly, Otx2 appears to determine the anterior identity that confers neurogenic potential of floor plate cells. Consequently, ectopic expression of Otx2 in the ventral hindbrain induces midbrain dopamine neurons, partly by inducing Lmx1a from floor plate cells, which normally do not give rise to neurons (Ono et al., 2007).

While Wnt1 expression is largely unaffected by Lmx1a loss-of-function, Lmx1b is a crucial regulator of Wnt1 expression in midbrain dopamine progenitors at later developmental stages (Deng et al., 2011). In addition to the role of canonical Wnt signaling in early specification, Wnt1 and Wnt3a increase neurogenesis and regulate the proliferation of Nurr1-positive midbrain dopamine precursor cells (Castelo-Branco et al., 2003). Likewise, disruption of canonical Wnt signaling leads to neurogenesis defects and perturbs the migration and segregation of midbrain dopamine neurons (Tang et al., 2009). Wnt2 is also involved in midbrain dopamine neurogenesis through activation of the canonical pathway (Sousa et al., 2010). Wnt5a increases the number of midbrain dopamine neurons by promoting the acquisition of a fully mature dopaminergic phenotype through upregulation of Pitx3 expression (Castelo-Branco et al., 2003). Wnt5a is also thought to control morphogenesis, dopamine progenitor cell division, cell cycle exit (Andersson et al., 2008) and to be involved in dopamine axon growth and targeting (Blakely et al., 2011).

RA also seems to play a role in midbrain dopamine neuron differentiation. Retinal dehydrogenase 1 (Raldh1), which converts retinaldehyde into RA, is expressed in the vental midbrain already at E9.5 (Wallen et al., 1999). Raldh1 (and thus the RA level) is transcriptionally regulated in the midbrain by Pitx3 (Chung et al., 2005; Jacobs et al., 2007). Deficiency in Pitx3 results in the selective loss of substantia nigra compacta dopamine neurons (Hwang et al., 2003). Maternal supplementation of RA could partially rescue SNc dopamine neuron degeneration in the Pitx3 knockout mice (Jacobs et al., 2007). Pitx3 appears to regulate genes in both an RA-dependent and an RA-independent manner, which could explain the partial, but not complete, rescue of SNc dopamine neurons after RA supplementation in Pitx3 knockout mice. Here, the Pitx3 downstream targets Vmat2, Dat, Raldh1, En1, En2, and Cck were unaffected by RA treatment (Jacobs et al., 2011).

Other morphogens and growth factors are important for survival and maturation of midbrain dopamine neurons. Members of the transforming growth factors beta (TGFβ) superfamily, bone morphogenetic proteins (BMPs) 2, 6, and 7 are expressed in the developing ventral midbrain and promote the survival of dopamine neurons in the rat (Jordan et al., 1997; Chou et al., 2008a,b). Furthermore, TGFβ 2-3, activin and glial cell line-derived neurotrophic factor (GDNF) are neurotrophic factors for dopmine neurons (Lin et al., 1993; Poulsen et al., 1994; Krieglstein et al., 1995a,b; Farkas et al., 2003; Roussa et al., 2006). GDNF appears to act as a target-derived neurotrophic factor through its high expression in striatal neurons that are innervated by SNc dopamine neurons (Hwang et al., 2003; Oo et al., 2005). In addition, GDNF is transiently expressed in the midbrain during dopamine neuron specification. Here, GDNF induces Pitx3 via NF-κ B-mediated signaling. Pitx3 is in turn required for activating the expression of brain-derived neurotrophic factor (BDNF) in a subpopulation of SNc dopamine neurons during embryogenesis. The loss of BDNF expression correlates with the increased apoptotic cell death of SNc dopamine neurons in the Pitx3 knockout mouse (Peng et al., 2011).

## Spinal motor neuron development

Motor neurons are the main regulators of movement, they extend axons that reach the periphery from the CNS and innervate skeletal and smooth muscles, forming neuromuscular junctions (NMJs). In the fatal neurodegenerative diseases amyotrophic lateral sclerosis (ALS) and spinal muscular atrophy (SMA), motor neurons degenerate, leading to progressive paralysis and respiratory failure. Motor neurons are found at different levels of the CNS, in the cortex, midbrain, hindbrain and spinal cord. Motor neurons present in the spinal cord can be divided into three subgroups: alpha (α), beta (β), and gamma (γ), depending on the type of muscle fiber they innervate. Muscle fibers can be intra or extrafusal according to their role in motor control. Extrafusal fibers are innervated by α motor neurons and generate contraction, while intrafusal fibers are innervated by γ motor neurons and serve in fine motor control and proprioception. β motor neurons are less well-described, but are known to innervate both fiber types. α motor neurons are the most abundant subtype present in the spinal cord and are known to display a larger size and more extensive connectivity within the spinal cord compared to γ motor neurons. In fact, α and γ motor neurons differ also in their pre-synaptic inputs and post-synaptic targets.

α motor neurons can be further subdivided into fast-twitch fatigable (FF), fast-twitch fatigue-resistant (FR), and slow-twitch fatigue-resistant (S), depending on the contractile properties of the muscle fibers they innervate (Burke et al., 1973). Importantly, these motor neuron groups show differential vulnerability to degeneration in ALS (Kawamura et al., 1981; Frey et al., 2000). Motor neurons differ in cell size based on their functional properties and the fiber type they innervate (Henneman et al., 1965; Kernell et al., 1999). For instance, the motor unit formation (muscle fibers innervated by the same motor neuron) has a hierarchical composition, and the size of the recruited motor neurons is correlated to the muscle unit force. For this reason, FF and FR motor neurons, that are characterized by higher force requirement, are larger in size than S motor neurons (Kernell et al., 1999). Slow and fast twitch motor neurons can be distinguished by a set of markers, at least during adulthood. For example, the expression of the synaptic vesicle protein SV2A is described to be restricted to S motor neurons (Chakkalakal et al., 2010). Moreover, oxidative enzymes showed greater cytoplasmic activity in smaller sized motor neurons, with higher levels of succinate dehydrogenase (SDH) being found in S motor neurons (Kernell et al., 1999). In contrast, fast twitch motor neurons presented higher levels of calcitonin gene-related peptide (CGRP). It has been suggested that CGRP has a role in connectivity maintenance, and thus is largely expressed in motor neurons presenting many terminal branches (Piehl et al., 1993; Kernell et al., 1999).

During development, motor neurons are grouped into columns and pools, acquiring a distinct topographic identity (Figure 1B). Motor neurons show different identities depending on the columnar group they belong to, and each column has a defined position on the rostro-caudal axis of the spinal cord. Four different columns, which innervate specific peripheral targets, can be found in the spinal cord. Lateral motor column (LMC) motor neurons are present at limb levels of the spinal cord and innervate limb muscles. The medial motor column motor neurons (MMC) innervating dorsal axial musculature are present at all levels of the spinal cord (Prasad and Hollyday, 1991; Gutman et al., 1993). The hypaxial motor column (HMC), also found at thoracic levels, is innervating intercostal and abdominal wall musculature. The preganglionic column (PGC) is composed of visceral motor neurons found at thoracic levels and innervating sympathetic ganglia (Figure 1B). LMC motor neurons are further divided into lateral motor neurons projecting dorsally to innervate extensor muscles, and medial motor neurons, projecting ventrally to innervate flexor muscles (Landmesser, 1978a; Tosney and Landmesser, 1985). Importantly, the segregation of motor neurons into motor columns correlates with the expression of distinct LIM-homeodomain transcription factors (Tsuchida et al., 1994) and Hox genes (Dasen et al., 2003, 2005, 2008).

Furthermore, motor neurons belonging to a given column are organized into pools. A motor pool consists of motor neurons that innervate a single muscle. Motor neurons display different positions inside the LMC column and innervate distinct target muscles depending on their topographic distribution (Landmesser, 1978b; Hollyday and Jacobson, 1990). In this respect, the LIM homeodomain protein Lhx1 directs dorsal LMC projections in the limbs, through the regulation of the EphA4 guidance receptor (Eberhart et al., 2002; Kania and Jessell, 2003). Moreover, Hox genes seem to be crucial both in LMC motor neuron pool identity determination and in the high precision of axon muscle targeting (Landmesser, 2001; Dasen et al., 2003).

Spinal motor neurons are generated through a specific gradient of extrinsic signals found in the embryo neural tube, acting along the dorso-ventral and rostro-caudal axis. Cells forming the ectoderm cell layer acquire an initial rostro-caudal and dorso-ventral identity mainly through the combined action of Shh and RA. Shh, as mentioned in section Dopamine and Oculomotor Neuron Development, is secreted from the notochord and the floor plate of the neural tube and is needed for ventralization (Ericson et al., 1996). Caudalization is regulated by a gradient of RA, produced initially by the presomitic mesoderm and somites (Maden, 2007) and is later on (around E11–12) found in the neural tube, all along the spinal cord (Horton and Maden, 1995) (Figure 2). BMP signaling is known to induce a dorsal identity, however, in combination with Shh, it was found to exert roles also ventrally. For instance, knock down of BMP in zebrafish embryos altered the localization of ventral neuronal subtypes (Barth et al., 1999; Nguyen et al., 2000). Neuronal progenitor response *in vitro* to Shh was found to be dependent on the BMP concentration (Liem et al., 2000). The patterning induced by Shh signaling activity promotes the expression of transcription factors characteristic of motor neuron progenitors, including the homeodomain transcription factors Pax6, Nkx6.1, and Nkx6.2 (Vallstedt et al., 2001) and the basic helix-loop-helix (bHLH) transcription factors Olig1 and Olig2 (Novitch et al., 2001; Zhou and Anderson, 2002). Moreover, during motor neuron generation (E9.5) cells start to express a defined pattern of transcription factors, including the homeobox gene Hb9 (Arber et al., 1999) and the LIM homeodomain genes Isl1 and Lhx3 (Ericson et al., 1992; Sharma et al., 1998). The expression of this group of transcription factors is crucial for motor neurons to acquire both capabilities of projecting axons to connect to muscles and releasing the neurotransmitter acetylcholine.

**Figure 3:**
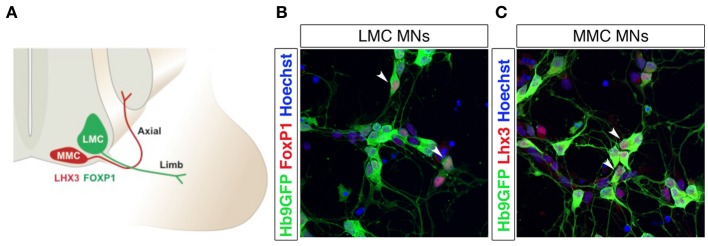
**Lateral and medial motor column motor neurons are distinguished by FoxP1 and Lhx3 expression**. Schematic drawing of lateral (LMC) and medial (MMC) motor column motor neurons innervating distal and proximal muscles, respectively **(A)**. Differentiation of Hb9-GFP mESCs into spinal motor neurons (MNs) using RA and SAG generates FoxP1^+^ LMC MNs **(B)** and Lhx3^+^ MMC MNs **(C)**. The arrow heads in panels **(B)** and **(C)** indicate motor neurons co-labeled with Hb9-eGFP and FoxP1 (in **B**) or Hb9-eGFP and Lhx3 (in **C**).

Two main transcription factors, Lhx3 and FoxP1, are used to discriminate between MMC and LMC motor neurons, *in vivo* and *in vitro* after induction of motor neurons from pluripotent stem cells (Figures 3A–C). Lhx3 is transiently expressed in all motor neuron progenitors, but is restricted to MMC motor neurons during later stages (Sharma et al., 1998). The maintained expression of Lhx3 renders motor neurons refractory to Hox gene patterning and the consequent segmentation into columnar identities (Tsuchida et al., 1994; Sharma et al., 2000; Dasen et al., 2003, 2005, 2008). Lhx3 expression and MMC identity is induced by the combined activity of Wnt4, Wnt5a, and Wnt5b, which are expressed in and around the floor plate. Increased levels of Wnt4/5 generated a majority of MMC motor neurons at the expense of LMC and HMC motor neurons, while depletion of Wnt4/5 reduced the number of MMC motor neurons (Agalliu et al., 2009). Forced expression of Lhx3 can reroute motor neurons and guide axons to proximal muscles (Sharma et al., 2000). FoxP1 is expressed in LMC motor neurons, and appears to interact with Hox proteins, as an accessory factor, to specify motor columnar identity, in a dose-dependent manner. Knock down of FoxP1 abolished LMC and MMC differentiation and reverted the spinal motor system to an ancestral state, lacking LCM motor neurons (Dasen et al., 2008), thus mimicing early aquatic vertebrate motor systems that contained only MMC and HMC motor columns (Fetcho and Reich, 1992). Loss of FoxP1 severely affected motor neuron connectivity patterns with limb axons appearing to select their projections and muscle targets at random (Dasen et al., 2008).

A second patterning system acting on the anteroposterior axis is triggered by a gradient of Fgf8, Gdf11, and RA, and induces the diversification of the already formed motor neurons (Liu et al., 2001; Dasen et al., 2003; Mazzoni et al., 2013). Gdf11 is a member of the TGFβ family, produced by the notochord and active at posterior levels. Fgf levels are also higher posterior, while RA exerts its action at anterior positions. This patterning induces the expression of Hox genes (Liu et al., 2001; Dasen et al., 2003, 2005, 2008), which are known to differ depending on the segmental level of the spinal cord (Carpenter, 2002). Specifically, lower levels of Fgf (from the presomitic mesoderm) induces the expression of Hox4–Hox8 genes at the cervical level of the spinal cord, while progressively higher levels of Fgf8 provokes Hox8–Hox9 and Hox10–Hox13 expression at thoracic and lumbar levels, respectively (Liu et al., 2001; Bel-Vialar et al., 2002; Dasen et al., 2003). These distinct Hox genes regulate motor column and pool identities. For instance, Hox6 determines cervical LMC motor neuron identity, while Hox10 specifies lumbar LMC motor neurons and Hox9 is needed for PGC neuron differentiation (Dasen et al., 2003; Shah et al., 2004; Wu et al., 2008). Misexpression of Hox genes can induce a motor column identity switch, with e.g., Hoxc9 expression at cervical levels switching the LMC to a PGC identity (Dasen et al., 2003). However, HMC and MMC columns are considered Hox-independent, due to their expression of Lhx3, which is a suppressor of FoxP1 and Hox genes in determining LMC/PGC fate. (Dasen et al., 2003, 2008; Rousso et al., 2008; Agalliu et al., 2009). Interestingly, the Wnt4/5-Lhx3 pathway seems to repress the Fgf-Hox pathway and vice versa (Agalliu et al., 2009). Hox gene regulation is highly complex, exemplified by that cervical LMC motor neurons express 11 different Hox genes (Hoxa3, a4, a5, a6, and a7; Hoxb7 and b8; and Hoxc4, c5, c6, and c8), some with distinct and others with co-segregated anterior-posterior expression domains within the motor column. As mentioned above, Hox genes play a fundamental role in LMC and PGC column differentiation, and exert their functions in combination with the cofactor FoxP1. PGC motor neurons show a Hox gene-dependent, lower level of FoxP1 than LMC motor neurons (Dasen et al., 2008). Phylogenetically, it seems that some of the more newly generated HMC neurons, which are devoid of Lhx3, became sensitive to Hox patterning, diverting from an HMC identity and turning into PGC and LMC motor neurons. Hox9 influence drives these cells to a PGC fate, with a consequent loss of Hb9 and acquisition of low levels of FoxP1. HMC motor neurons, instead, are devoid of FoxP1 and express Hb9. LMC motor neurons, patterned by Hox6 (cervical) and Hox10 (lumbar) activity, can express high levels of FoxP1 without Hb9 repression (Dasen et al., 2008). In addition, as previously explained, MMC and HMC motor neurons are specified by the LIM-homeodomain factor Lhx3 (Sharma et al., 1998), while FoxP1 expression is repressed here (Dasen et al., 2003) (Figure 3). In addition to controlling motor pool identities, Hox genes regulate motor neuron axon guidance and muscles-nerve connections. However, target path finding depends on extrinsic signals (trophic factors) provided by the mesoderm and muscles, which induce expression of the ETS transcription factors Pea3 and Er81 (Lin et al., 1998; Arber et al., 2000; Haase et al., 2002). Interestingly, these transcription factors are not present in all motor neuron pools, and their expression was shown to further induce pool organization after motor neurons have reached their target organs, both in terms of muscle specific innervation and motor-sensory connectivity and coordination (Livet et al., 2002).

## Utilizing embryonic stem cells to model development and disease

**Figure 4:**
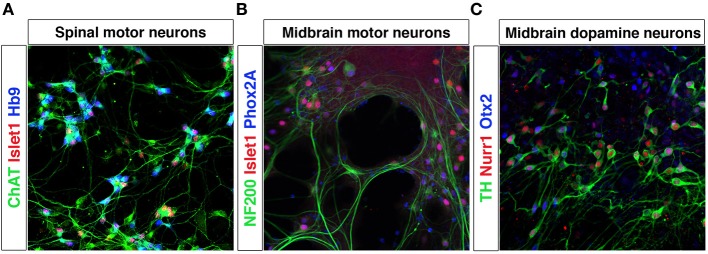
**Differentiation of mESCs into spinal motor neurons and midbrain neurons**. Spinal motor neurons (MNs) were generated from Hb9-GFP mESCs using RA and SAG **(A)**. MNs express Islet-1, have a healthy appearance and extend processes. Exposure of mESCs to SAG and Fgf8 generates midbrain motor neurons **(B)** that express NF200, Islet-1/2 and Phox2A, and grow in clusters and extend neurites and dopamine neurons **(C)** that express TH, Nurr1, and Otx2.

Pluripotent stem cells allow unlimited expansion and derivation of any cell type and thus represent an excellent source of *in vitro* generated neurons. The processes of neuronal fate specification can be recapitulated *in vitro* using pluripotent stem cells derived from ESCs (Evans and Kaufman, 1981; Martin, 1981) or iPSCs (Takahashi and Yamanaka, 2006). As in the embryo, Shh and RA specify motor neuron fate from pluripotent stem cells (Wichterle et al., 2002; Hu and Zhang, 2009). Dopamine neurons (Lee et al., 2000; Kriks et al., 2011) and oculomotor neurons can be generated by a combination of Shh, Fgf8, and Wnt (Figures 4A–C). iPSCs from patients with e.g., Parkinson disease or motor neuron diseases, including ALS and SMA (Demos et al., 2008; Park et al., 2008; Corti et al., 2012; Sanchez-Danes et al., 2012; Alami et al., 2014), are vital tools to study presymptomatic and symptomatic disease events in a dish. *In vitro* utilization of morphogens for the derivation of particular neurons typically results in the generation of multiple cell types, which are normally defined at the intersection of these signals. Thus, the desired neuronal lineage will constitute only a fraction of the cells. Further lineage restriction from stem cells can be accomplished by forced expression of specific transcription factors in a permissive environment, reviewed below in the section Forced Expression of Transcription Factors to Induce Neuronal Lineages. Alternatively, cell-sorting strategies allow for the selection of neuronal progenitors or mature neurons of a specific lineage, reviewed in the section Cell Sorting Strategies Allowing for Selection of Neurons.

## Forced expression of transcription factors to induce neuronal lineages

**Figure 5:**
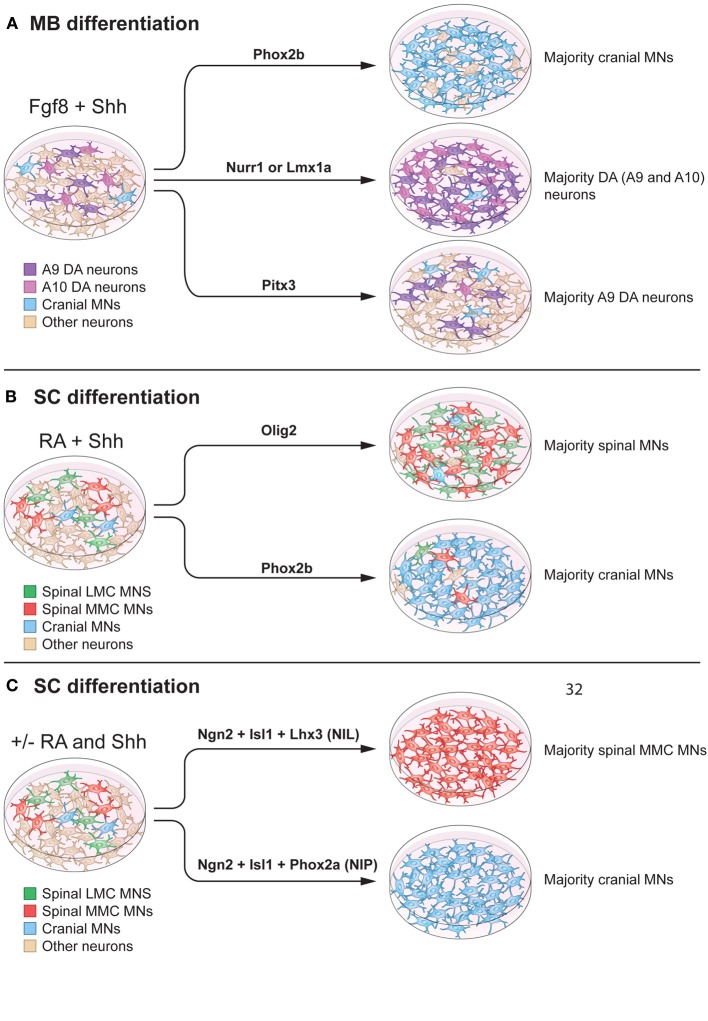
**Modulation of intrinsic determinants can promote induction of midbrain neurons and spinal motor neurons. (A)** Shh and Fgf8 induce midbrain neuron differentiation from stem cells. Forced expression of specific transcription factors, in a permissive environment, can further promote induction of specific neuronal lineages. Here we specifically summarize effects on mouse embryonic stem cells. For example, Phox2b can increase the percentage of cranial motor neurons (MNs) (to 90% of the neurons in the culture). Nurr1 and Lmx1a can induce dopamine neuron fate (to 80% of the neurons in the culture). Pitx3 can specifically promote an A9 dopamine (DA) neuron fate without affecting the total number of dopamine neurons in culture (25% of the neurons in the culture). **(B)** RA and Shh can induce motor neuron differentiation from pluripotent stem cells, with 15–30% of all cells adopting a motor neuron fate. Over-expression of Olig2 can increase the proportion of spinal motor neurons, without a preference for LMC or MMC motor neurons (90% of the neurons in culture). Forced expression of Phox2b induces a cranial motor neuron fate (90% of the neurons in culture). **(C)** mESCs can be directly converted into spinal motor neurons in the absence of Shh and RA, using forced expression of Ngn2, Isl1, and Lhx3 (NIL) (>99% of the neurons in culture), or into cranial motor neuron using Ngn2, Isl1, and Phox2a (NIP) (>99% of neurons in the culture).

Forced expression of transcription factors that determine specific neuronal fates *in vivo* can further induce lineage restriction from pluripotent stem cells. Generation of one cell type only could be beneficial for cellular therapies in degenerative diseases, for *in vitro* drug screening purposes and to study cell intrinsic mechanisms. Toward the goal of developing stem cell based therapies for Parkinson disease, great efforts have been made to generating highly enriched dopamine neuron cultures. Forced expression of transcription factors, including Nurr1 (NR4A2), Lmx1a and Pitx3, in combination with Shh and Fgf8 signaling, can promote dopamine neuron differentiation (Figure 5A). Nurr1 over expression increased the proportion of dopamine neurons from 25 to 80% of all neurons. Specifically, Nurr1 up-regulated a dopamine neurotransmitter phenotype, with increased expression of TH, DAT, AADC, and c-ret (Chung et al., 2002; Kim et al., 2002). Pitx3 did not increase the total number of dopamine neurons, but shifted the fate preferentially into SNc-like dopamine neurons, defined by Raldh1 expression (Chung et al., 2005) (Figure 5A). Lmx1a over expression in nestin^+^ cells could trigger dopamine neuron differentiation, by inducing Msx1 and Ngn2 expression, with approximately 80% of neurons showing a correct phenotype (Andersson et al., 2006; Friling et al., 2009; Panman et al., 2011). As Lmx1a is also expressed in the roof plate, and a determinant of dorsal cell fates, a ventral environment was necessary for induction of a dopamine neuron fate (Andersson et al., 2006).

Cranial and spinal motor neuron fates can be induced by over expression of Phox2a, Phox2b or Olig2 in neuronal precursors, in combination with morphogens (Figure 5A). Specifically, cranial motor neuron differentiation was promoted by Phox2b, in combination of Shh and Fgf8 or Shh and RA, to ~90% of all neurons (Panman et al., 2011) (Figures 5A,B). If over expression of Phox2b was instead performed in the presence of BMP7 and Fgf8, noradrenergic neurons were generated. Phox2a, in the presence of Shh and Fgf8, favored a cranial motor neuron fate (Mong et al., 2014), while Olig2 in the presence of Shh and RA, increased the proportion of spinal motor neurons to ~90% of all neurons.

It has been reported that spinal motor neurons derived from mESCs, using RA and a Shh agonist (Hh-Ag1.3 or SAG) showed electrophysiological properties and transcription factor expression typical of MMC motor neurons (Wichterle et al., 2002; Soundararajan et al., 2006). A recent study demonstrated that the combination of two smoothened (Smo) agonists, SAG and purmorphamine, could increase the proportion of LMC motor neurons in culture, compared to Shh. The effects of SAG and purmorphamine alone were not analyzed (Amoroso et al., 2013), thus precluding a conclusion as to the individual or synergistic effects of the agonists. In our hands SAG yields both LMC and MMC motor neurons, defined by the presence of Lhx3^+^ and FoxP1^+^ (Figures 3B,C). It would be of major interest to perform large-scale chemical screens to further identify compounds that can regulate the above-discussed intrinsic determinants, which are known to shift neuronal identities *in vitro*.

Several studies have focused on using multiple transcription factors to achieve synergistic promotion of neuronal maturation. The combination of Ngn2, Islet-1, and Phox2a (NIP) induced cranial motor neuron fate from mESCs more robustly (>95% of all neurons) than Phox2a alone. This work demonstrated that transcription factors can work in synergy to induce desired neuronal lineages with high efficiency. Ngn2, Islet-1, and Lhx3 (NIL) accelerated mouse and human ESC differentiation into spinal motor neurons (Hester et al., 2011; Mazzoni et al., 2013). Importantly, NIL factors could promote spinal motor neuron differentiation, even in the absence of RA (Figure 5C). However, the lack of RA decreased Hox4, Hox5, and Hox6 gene expression, while increasing rostral neural markers. When NIL forced expression was combined with RA administration, Hox gene expression was re-established, confirming the fundamental role of RA in patterning cervical motor neurons. Furthermore, the NIP and NIL factors appeared to directly program pluripotent stem cells into neurons, determined by lack of progenitor markers Olig1 and Olig2 and the presence of the post-mitotic marker Hb9 (Mazzoni et al., 2013).

Forced expression of transcription factors can also be used to directly convert fibroblasts into neurons. Specifically, introduction of Asc1, Brn2, and Myt1l (BAM) into mouse fibroblasts was shown to induce neuron generation (Vierbuchen et al., 2010). The BAM factors could directly reprogram human pluripotent stem cells (Pang et al., 2011) and fibroblasts (Pfisterer et al., 2011) to neurons, but the efficiency of human fibroblasts conversion improved greatly by the addition of the bHLH transcription factor NeuroD1 (Pang et al., 2011). Direct conversion of fibroblasts into dopamine neurons and motor neurons requires the addition of lineage specific fate determining factors. Dopamine neurons could be induced when BAM factors were combined with Lmx1a and FoxA2 (Pfisterer et al., 2011). However, the combination of the transcription factors, Ascl1, Nurr1, and Lmx1a appeared more efficient for direct reprogramming of fibroblasts into dopamine neurons (Caiazzo et al., 2011). Generated dopamine neurons expressed TH, Raldh1, VMAT2 and showed appropriate electrophysiological properties. Here, also Parkinson disease patient fibroblasts were reprogrammed (Caiazzo et al., 2011). The combination of the BAM factors with transcription factors known to determine motor neuron fate, including Lhx3, Hb9, Isl1, and Ngn2, converted mouse fibroblasts into motor neurons. Moreover, the addition of NeuroD1 allowed for motor neuron differentiation from human fibroblasts (Son et al., 2011).

The studies reviewed clearly demonstrate the utility of using forced expression of transcription factors to direct specific neural fates in a permissive environment *in vitro*. The resulting enriched cultures are beneficial for determining cell intrinsic properties of distinct neuron groups, drug-screenings, disease-modeling and for transplantation.

## Cell sorting strategies to enrich for neurons

While forced expression of transcription factors can efficiently drive specific neuronal lineage, commonly the culture still contains a mixture of cells, including pluripotent cells, progenitors and several neuronal types. For both *in vitro* and *in vivo* purposes, the inclusion of actively dividing cells can cause severe problems. Remaining proliferating cells can quickly become the major cell type with long-term culturing of stem cell-derived neuronal cultures (Figure 7). Furthermore, it is unlikely that the proliferating cells will differentiate into the desired neuronal phenotype(s). Thus, the culture will be continuously diversified unless the patterning phase is maintained. For transplantation purposes the inclusion of dividing cells can be detrimental due to the potential formation of tumors or teratomas (Bjorklund et al., 2002). Removal of unwanted proliferating cells is relatively straightforward using cell sorting strategies such as magnetic activated cell sorting (MACS) or fluorescent activated cell sorting (FACS). Mouse embryonic and adult stem cells express stage specific embryonic antigen-1 (SSEA-1), while human stem cells express SSEA-3 on their surface (Solter and Knowles, 1978; Capela and Temple, 2002). Removing proliferating SSEA-1^+^ cells from stem cell-derived midbrain neuronal cultures using FACS can avert tumor formation after transplantation (Hedlund et al., 2007, 2008; Wernig et al., 2008; Ganat et al., 2012). It is vital to emphasize that even the inclusion of small numbers of pluripotent stem cells can be detrimental. Indeed, transplantation of cellular suspensions containing only a fraction of SSEA-1^+^ cells (<1% SSEA-1^+^ cells) sometimes give rise to tumors (Hedlund et al., 2008; Ganat et al., 2012).

The inclusion of multiple neuronal lineages could result in the lack of a functional outcome, aberrant results or assays with a complicated readout. Therefore, it can be advantageous to enrich for a neuronal cell type of interest, in addition to removing unwanted proliferating cells (Figure 7). For example, the accidental inclusion of serotonergic neurons in transplanted Parkinson disease patients is considered partly responsible for fetal graft-induced dyskinesia and L-DOPA induced dyskinesia (Carlsson et al., 2009; Politis et al., 2010; Bezard et al., 2013). Serotonin neurons can convert L-DOPA into dopamine and synaptically release it. However, they lack an appropriate feedback control (no dopamine transporter), which leads to over-stimulation of post-synaptic dopamine neurons onto striatal neurons. Indeed, fetal dopamine neuron grafts in Parkinson patients have been shown to contain serotonergic neurons (Mendez et al., 2005, 2008; Politis et al., 2010) and these neurons appeared to hyper innervate the patient striatum (Politis et al., 2010).

Several strategies have been used to enrich for dopamine progenitors or mature dopamine neurons from fetal tissues and from stem cell-derived cultures. The initial studies which proved that primary dopamine neurons could be enriched by FACS, used either NSP-4 based antibody labeling (di Porzio et al., 1987), dye injection (Kerr et al., 1994) or transgenic fluorescence based on TH expression (Sawamoto et al., 2001; Donaldson et al., 2005). Such enriched dopamine neurons could survive *in vivo* after transplantation and induce partial functional recovery in 6-hydroxydopamine lesioned Parkinsonian rats (Sawamoto et al., 2001; Donaldson et al., 2005). Purification of midbrain dopamine neurons derived from pluripotent stem cells has proven more challenging than enrichment from primary tissue. Specifically, stem cell-derived cultures are rarely synchronized temporally or spatially and will therefore simultaneously contain cells of diverse developmental stages and cell fates. Thus, if a marker is expressed in several different cell types during development it will not be particularly suitable for sorting. TH for example, which labels catecholaminergic neurons, including midbrain dopamine neurons, is transiently expressed in multiple lineages during development, including cells with proliferative capacity (Lindeberg et al., 2004). Therefore, when the TH promoter was used to drive GFP expression in stem cell-derived midbrain cultures, dopamine neurons were labeled, as well as proliferating cells. To acquire an enriched neuronal culture it was necessary to combine the TH-GFP reporter with a negative selection step for SSEA-1 to remove proliferating GFP^+^ cells (Hedlund et al., 2007). Pitx3 is a more restricted marker for midbrain dopamine neurons than TH. While Pitx3 is transiently expressed in skeletal muscle and the lens of the eye (Smidt et al., 2004; Zhao et al., 2004), those cell types are typically not generated during *in vitro* differentiation of stem cells toward a midbrain fate (Hedlund et al., 2008). Transplantation of FACS-enriched Pitx3-eGFP ESC-derived dopamine neurons into Parkinsonian rats, reversed drug-induced rotational behavior and the transplanted neurons innervated the host striatum (Hedlund et al., 2008; Ganat et al., 2012).

However, FACS-enriched cell suspensions, containing ≥80% Pitx3-GFP^+^ neurons, infrequently gave rise to tumors due to the presence of SSEA-1^+^ proliferative cells. If the cells were subjected to a second round of FACS, up to 98% dopamine neurons were retrieved, and these cells survived *in vitro* when plated on astrocytes (Hedlund et al., 2008) or matri gel (Ganat et al., 2012). These dopamine neurons also survived *in vivo* and reversed amphetamine-induced rotational behavior without forming tumors. However, the survival of the cells was affected, either from mechanical injury caused by the second sorting step or through the exclusion of cell types that provided trophic support (Ganat et al., 2012). To discriminate between these two options, double-sorted neurons should be transplanted alone or in combination with the negative fraction that was removed between the first and second FACS.

Sorting and transplanting mature dopamine neurons derived from pluripotent stem cells is clearly feasible, but the efficiency is relatively low. FACS and transplantation of dopamine neurons at the stage of cell cycle exit, using a Nurr1-GFP reporter, resulted in more robusts grafts than a Pitx3-GFP reporter. While neuronal progenitor selection using a Hes5-GFP reporter line, resulted in grafts that contained a majority of non-dopamine neurons (Ganat et al., 2012). This indicates the timing of selection and transplantation is of importance and that very early markers might be less beneficial than later ones. The floor plate marker Corin has been used to isolate ventral midline cells, from the developing midbrain, which co-expressed the dopamine neuron marker Lmx1a (Ono et al., 2007). However, the broad anterior-posterior expression domain of Corin makes it less suitable for selecting midbrain progenitors from pluripotent stem cells that are difficult to restrict spatially.

Importantly, it is the SNc (A9) dopamine neurons that innervate the dorsolateral striatum after transplantation in Parkinson disease models and induce functional recovery. VTA (A10) dopamine neurons mainly appear to project to the frontal cortex and other forebrain areas (Thompson et al., 2005; Grealish et al., 2010). Thus, it is not sufficient to enrich for dopamine neurons, but specific cell-surface markers or genetic labeling to distinguish SNc and VTA are needed. In this regard, the expression of the G-protein inward rectifying potassium channel subunit 2 (Girk2, Kir3.2) was previously considered a specific marker of vulnerable SNc dopamine neurons (Mendez et al., 2005; Thompson et al., 2005; Lammel et al., 2008). However, a detailed study of the human midbrain revealed that Girk2 expression levels were similar in the ventral and dorsal tiers of the human SNc (Reyes et al., 2012), with 77% of SNc and 55% of VTA neurons showing a strong Girk2 immunoreactivity. The proportion of TH neurons showing colocalization with Girk2 was similar in the mouse brain (Fu et al., 2012; Reyes et al., 2012). Therefore, the most reliable criterion to separate SNc and VTA dopamine neurons *in vitro* appears to instead be the absence of calbindin-D28k in SNc dopamine neurons (German et al., 1992; Damier et al., 1999a). Nonetheless, it should be noted that around 12% of human and 20% of mouse pars medialis SNc dopamine neurons co-express calbindin-D28k (Reyes et al., 2012).

**Figure 6:**
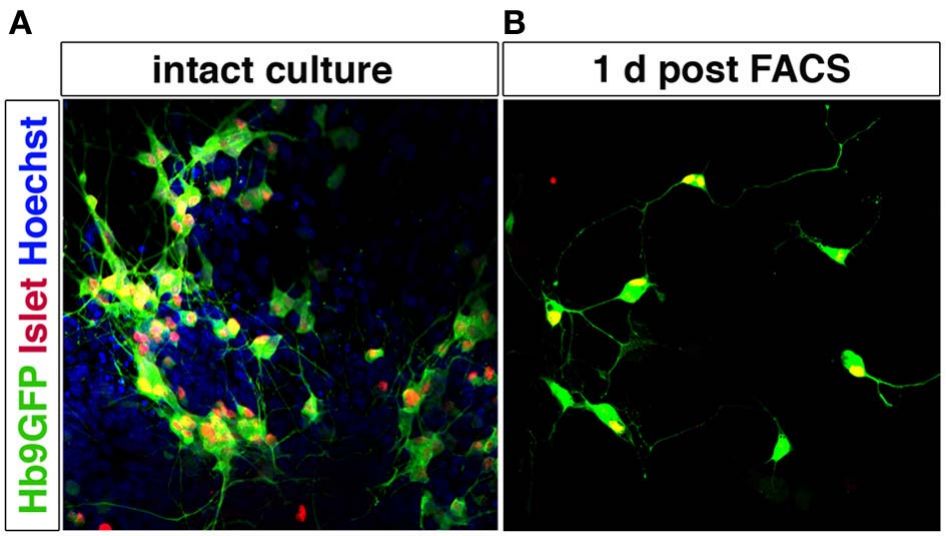
**Motor neurons can be purified by FACS using Hb9-GFP expression**. Spinal motor neurons derived from Hb9-GFP mESCs extend processes and express Islet-1/2. Hoechst staining of all nuclei shows that the intact culture contains cells other than motor neurons (**A**). Hb9-GFP motor neurons enriched by FACS show a healthy appearance with extended processes 1 day post sorting (**B**).

Motor neurons can be purified using MACS and FACS. p75^NTR^ has been used to isolate motor neurons from the developing spinal cord using immunopanning (Camu and Henderson, 1992; Wiese et al., 2010) or MACS (Arce et al., 1999). FF, FR, and S motor neuron populations have been isolated (for gene expression profiling without further culturing) by FACS following injection of retrograde tracers into muscle (Saxena et al., 2009). Corticospinal motor neurons have also been purified using FACS, based on dye injections *in utero* (Arlotta et al., 2005; Ozdinler and Macklis, 2006). mESC-derived motor neurons can be enriched by FACS using the Hb9 promoter to drive eGFP expression (Wichterle et al., 2002) (Figure 6). This selection approach has also been successfully applied to human motor neurons derived from ESCs (Di Giorgio et al., 2008; Marchetto et al., 2008) and from induced pluripotent stem cells (Corti et al., 2012). Hb9 shows a high specificity for motor neurons in the nervous system, although there are smaller populations of Hb9^+^ interneurons, as shown both in Drosophila (Odden et al., 2002) and mouse (Wilson et al., 2005). Hb9 is also expressed outside the nervous system, including the pancreas, but no non-neuronal Hb9^+^ cells appear to be generated during differentiation of pluripotent stem cells into motor neurons using RA and Shh. Genetic labeling could allow for isolation specifically of trunc- (MMC) or limb-innervating (LMC) motor neurons based on Lhx3 or FoxP1 expression, respectively, in combination with a Hb9-GFP reporter. As Lhx3 is broadly expressed in motor neurons during early development, the presence or lack of FoxP1 would be a the more reliable discriminatory marker.

Motor neurons, isolated by FACS using the Hb9-GFP reporter survive *in vitro* when supplemented with a combination of growth factors, including GDNF, BDNF, CNTF, and NT3 (Wichterle et al., 2002). Hb9^+^ motor neurons also survive *in vivo* after grafting and connect with target muscles (Wichterle et al., 2002; Harper et al., 2004; Gao et al., 2005; Deshpande et al., 2006; Bryson et al., 2014), and induce functional recovery in motor neuron lesion models (Deshpande et al., 2006). However, published studies up to date have transplanted unsorted ESC-derived motor neurons. FACS-purified motor neurons will presumably show lower survival rate *in vivo*, but that remains to be investigated. Recent experiments showed that both astrocytes and muscle secrete factors that promote survival of motor neurons *in vitro*. However, astrocyte-conditioned media saved substantially more motor neurons than muscle-conditioned media and only astrocyte-secreted factors could rescue motor neurons after unilateral limb bud removal (Taylor et al., 2007). These data indicate that co-transplantation of purified motor neurons with astrocytes could be beneficial for graft survival. Alternatively, stem cells can be genetically engineered to over express growth factors to trophically support generated MNs (Bryson et al., 2014).

Furthermore, pan-neuronal sorting strategies have utilized the cell surface expression of PSA-NCAM to enrich for neurons (Schmandt et al., 2005; Panman et al., 2011). As astrocytes can express PSA-NCAM (Minana et al., 1998) this approach could enrich for a mixture of neurons and glia. PSA-NCAM is also expressed on other non-neuronal cells, including beta cells (Kiss et al., 1994; Bernard-Kargar et al., 2001). However, it is unlikely that pancreatic cells, formed from definite endoderm (D'Amour et al., 2005) are generated by the conditions used to derive midbrain and spinal neurons.

Ideally specific neuronal populations can in the future be isolated using a combination of two to three cell surface markers, which would bypass the need for viral vectors or genetic engineering of stem cells for labeling.

## Structured heterogeneity to study development and disease in a dish

Modeling development, disease and prospective therapies in a dish could benefit enormously from systems displaying structured heterogeneity, where desired neurons are appropriately connected pre-and post-synaptically. Ideally, *in vitro* systems should be complex enough to reflect vital processes of development and disease, yet simple enough to be reproducible, and therefore likely would benefit from some reductionism. By modulating the extrinsic environment, and thus intrinsic determinants, it should be possible to accomplish directed sequential generation of neural progenitors within a domain, followed by self-organization and synaptic establishment to create a reductionist model of that brain region.

Importantly, mouse and human pluripotent stem cells have been successfully used to generate self-organizing three-dimensional structures that resemble the cortex (Eiraku et al., 2008; Gaspard et al., 2008; Lancaster et al., 2013). These “organoids” could be used both to study human brain development and disease, as exemplified by modeling of microcephaly using iPSCs (Lancaster et al., 2013). Other highly complex systems, such as the optic cup structure has also been formed through self-organization in human ESC culture, with the retina growing into a multilayered tissue containing both rods and cones (Nakano et al., 2012). This studies point out the remarkable possibility to reconstruct development and generate specific structures *in vitro* from pluripotent stem cells.

While no studies so far have generated a three dimensional midbrain structure *in vitro*, the existing induction protocols generate several neuronal types that are present in the midbrain/hindbrain, including dopamine neurons, oculomotor neurons (Figures 4B,C) and serotonin neurons (Lee et al., 2000; Kim et al., 2002). Interestingly, transplantation experiments using primate ESC-derived neurons differentiated toward a midbrain fate revealed that ESC-derived dopamine neurons appropriately innervated ESC-derived striatal neurons (DARPP32^+^ and Bf-1^+^) within the graft (Ferrari et al., 2006) (Figure 8A). The target innervation was specific as other neurons within the graft, including Nkx2.1^+^ ganglionic eminence neurons, Nkx2.2^+^ pallidal and diencephalic progenitors or Pax6^+^ dorsal telencephalic progenitors did not appear innervated (Ferrari et al., 2006). This clearly shows that despite the diverse cellular composition of the graft, ESC-derived dopamine neurons displayed an appropriate target connectivity and functionality. It also shows that an induction protocol used to generate dopamine neurons can generate their striatal target neurons. While this could be a disadvantage in transplantation experiments, where grafted neurons mainly innervate targets within the graft rather than the host, it is advantageous for *in vitro* experimentation where a self-organizing circuitry could be generated within a dish. Further experimentation is required to determine if a complete circuitry, including GABAergic globus pallidus neurons (Figure 8A) are also generated in the Shh, Fgf8 and Wnt-based induction protocols or if modulations are needed to accomplish this.

**Figure 7:**
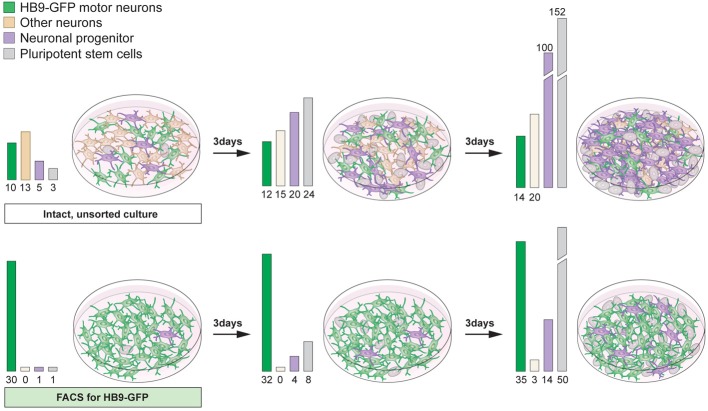
**Proliferative cells can become the main component of differentiated stem cell cultures with time**. Differentiation of pluripotent stem cells using RA and Shh results in the generation of motor neurons (15–30%) and interneurons, particularly V0 and V1. The culture will also contain neuronal progenitors and pluripotent stem cells (**top panel**). If the intact culture is maintained, after differentiation, proliferating cells will soon become the main component (**top panel**). Enrichment of motor neurons, using FACS for the Hb9-GFP reporter, results in cultures mainly composed of motor neurons, enabling an analysis of motor neuron specific properties and functions (**bottom panel**). However, with time, if cultures are not mitotically inhibited, sorted cultures will contain an increasing number of proliferating cells, since FACS rarely results in 100% enrichment (**bottom panel**).

**Figure 8:**
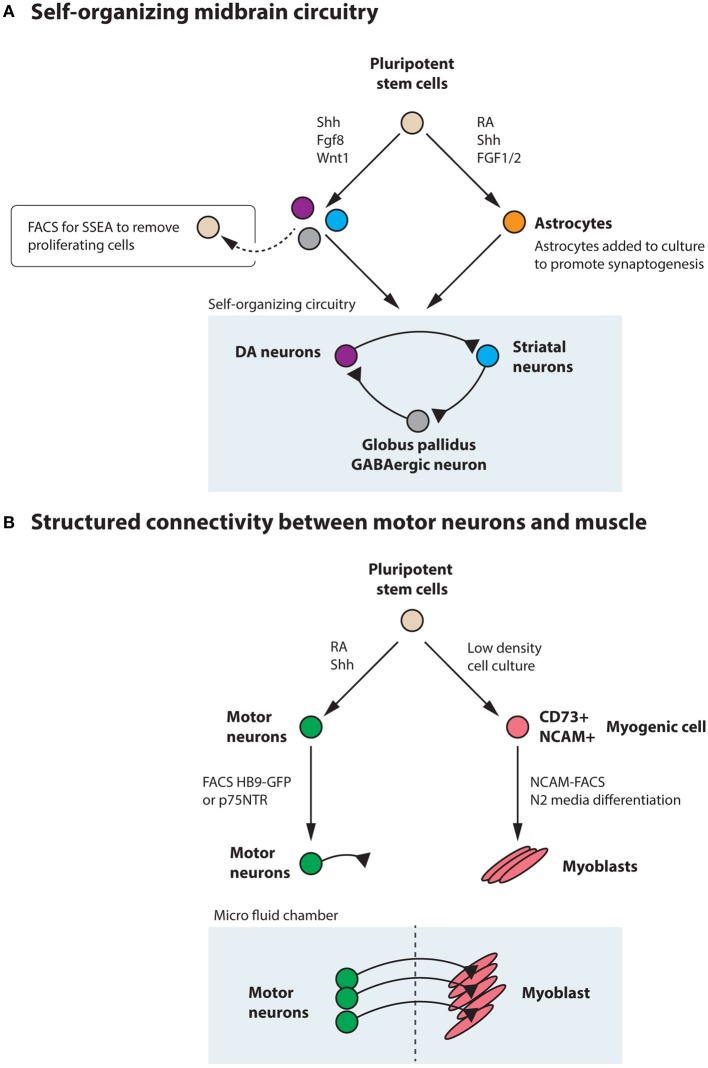
**Pluripotent stem cells could be utilized to generate a self-organizing midbrain circuitry or structured connectivity between motor neurons and muscle. (A)** Utilizing the morphogens Shh, Fgf8, and Wnt1, pluripotent stem cells generate dopamine neurons, striatal neurons, and GABAergic neurons that could form a self-organizing circuitry during appropriate conditions. Remaining pluripotent stem cells could be removed by FACS for SSEA. Astrocytes, generated from stem cells through the action of RA, Shh, and FGF1/2 could be added to the culture to promote synaptogenesis. **(B)** Motor neurons can be generated from pluripotent stem cells by the addition of RA and Shh and further enriched through FACS for Hb9-GFP or the cell surface marker p75^NTR^. These motor neurons can be appropriately connected to muscle, derived from stem cells through e.g., low-density culture, enrichment for CD73^+^NCAM^+^ myogenic progenitors and subsequent differentiation in N2 media. Culturing in a microfluidics chamber ensures that connectivity is appropriately achieved and that flow between the compartments can be regulated.

Generating a circuitry for motor neurons based on ESC-derived cells is naturally more complex since motor neurons synapse onto muscle outside the CNS. Here, it will likely be necessary to generate muscle and motor neurons separately, since the induction protocols are quite distinct. Commonly, the inductive spinal motor neuron protocols (and midbrain neuron protocols as well) utilize an initial step called embryoid body (EB) formation, where floating spheres are generated, which can form all three germ layers. Here, a neuroectoderm fate is enforced, at the expense of mesoderm and endoderm formation, with the imposed action of RA and Shh to drive the generation of neurons. As discussed above, these inductive protocols do not only generate spinal motor neurons, but also other cell types, including several distinct interneuron populations, that are born in the intersection of these two morphogens (Wichterle et al., 2002) (Figure 7). With time, glia progenitors also appear and mature into astrocytes. Thus, this EB based differentiation, mimics normal neural development and in sorts generates a “mini” spinal cord containing all the appropriate cell types. The main thing this system is lacking, is an appropriate connection to the periphery. If these EBs are attached, the motor neurons generated within, extend long distally projecting axons. When the generated motor neurons are plated directly onto muscle, they form appropriate connections with their targets through NMJs (Corti et al., 2012). However, to ensure that motor neurons and muscle are appropriately connected, it will be beneficial to use microfluidics devices where motor neurons and muscle cell bodies are kept separate and motor axons can connect to muscle through narrow channels. Thus, the only connection is through the motor axon and the muscle. Here the flow can be directed so that factors secreted by either compartment can have more or less influence on the other compartment (Figure 8B). While partly dissociated EBs could be fitted into such microfluidics devices, it could be beneficial to isolate motor neurons by FACS (Figure 6) or MACS using transgenic expression of Hb9-GFP or cell surface expression of p75^NTR^ (Figure 8B). This would likely ensure that motor neurons perform less targeting within the EB and instead grow to the distal muscle target. Skeletal muscle has been successfully generated from human ESCs and iPSCs (Barberi et al., 2007; Darby et al., 2012; Hosoyama et al., 2014). The induction protocols used include; using low density cell culture and selection for CD73^+^ myogenic cells based on NCAM^+^ cell surface expression and further differentiation (Figure 8B) (Barberi et al., 2007); conditional expression of Pax7 to derive myogenic progenitors (Darby et al., 2012) and a sphere-based culture system where FGF2-conditioned expansion induced myogenic progenitor generation (Hosoyama et al., 2014). Such a NMJ system will be highly beneficial for studying effects on axonal transport, both retrograde and anterograde. If muscle or motor neurons were derived from iPSCs from ALS patients, it could enable an analysis of how one cell with disease affects a healthy counterpart. Here, we could also study the role of interneurons in disease pathogenesis in ALS, by adding disease-interneurons to a healthy motor neuron-muscle circuitry or vice versa. Spinal interneurons are, as previously mentioned, generated simultaneously with motor neurons when pluripotent stem cells are exposed to Shh and RA. These interneurons, derived from healthy individuals or motor neuron disease patients, could be enriched for by removing remaining pluripotent stem cells (SSEA^+^) and motor neurons (p75^NTR+^). Any glial progenitors present would be in a clear minority and could be mitotically inhibited by the addition of e.g., AraC. Furthermore, to better understand neuronal vulnerability and protection in Parkinson disease and ALS, which are axonopathies, it would be very valuable to study the connectivity with the target cells. Hence, *in vitro* modeling of Parkinson disease could benefit from keeping dopamine neurons and their striatal targets in separate compartments, through microfluidics devices, to better visualize early axonal dysfunction. Here, dopamine neurons, could be enriched by FACS using a Nurr1-GFP or Pitx3-GFP reporter (Hedlund et al., 2008; Ganat et al., 2012) and cultured in one compartment, while their dorsolateral striatal targets, could be enriched for by e.g., Dopamine D1 receptor, Drd1a, expression (Gerfen et al., 1990; Lobo et al., 2006; Ena et al., 2013) and grown in the other compartment. For *in vitro* drug screening purposes, being able to quantify loss of connectivity, for example of the NMJ or dopamine neuron-striatum, rather than analyzing neuronal loss would improve the understanding of the mechanisms of action of particular drugs and their usability *in vivo*.

In summary, specific neuronal fates are determined through the interplay between extrinsic morphogen signals and intrinsic determinants. These processes can be recapitulated when directing neurogenesis from pluripotent stem cells in a dish. Utilizing morphogen signals to derive neurons from stem cells *in vitro*, e.g., Shh and Fgf8 to generate dopamine neurons or Shh and RA for motor neuron induction, will lead to the generation of a repertoire of cell types, which are normally generated at the intersection of the used signals. If morphogen signals are combined with forced expression of transcription factors that are lineage determinants, specific neuron types can be highly enriched for. A similar end result can be achieved using FACS or MACS to enrich for the desired neuronal types. Here, cell specific surface markers are required or genetic engineering to label only the desired cells for the selection procedure. However, acquiring cultures containing one cell type only typically necessitate a negative selection step, to remove cells with proliferative capacity, or treatment with agents inhibiting mitosis. If neurons are to be used for cellular therapies, it is vital to utilize cultures containing only the desired cell type(s), for functionality, efficacy and safety reasons. It could also be advantageous in the study of cell intrinsic properties and in drug screens. However, *in vitro* systems displaying structured heterogeneity, where neurons are appropriately connected, pre- and post-synaptically, representing a reductionist model of that brain region, could be superior for studying development and disease in a dish. Such systems can sometimes be acquired through exposing pluripotent stem cells to morphogenic signals followed by self-organization *in vitro*. It should also be possible to construct simplified circuitry systems by artificially combining selected cell types, such as motor neurons and muscle that are generated in distinct induction protocols.

### Conflict of interest statement

The authors declare that the research was conducted in the absence of any commercial or financial relationships that could be construed as a potential conflict of interest.
